# Supplementary material for: Asymmetric elastoplasticity of stacked graphene assembly actualizes programmable untethered soft robotics
Source: Nat Commun. 2020 Aug 31;11:4359. doi: 10.1038/s41467-020-18214-0 (PMC7459344; doi:10.1038/s41467-020-18214-0)
Supplement: Supplementary file 1 — Supplementary Information [file 41467_2020_18214_MOESM1_ESM.pdf]

Supplementary Information for

Asymmetric elastoplasticity of stacked graphene assembly actualizes programmable untethered soft robotics

Wang et al.

Supplementary Note 1. Theoretical prediction of the curvatures of SGA/PE films after constrained tempering

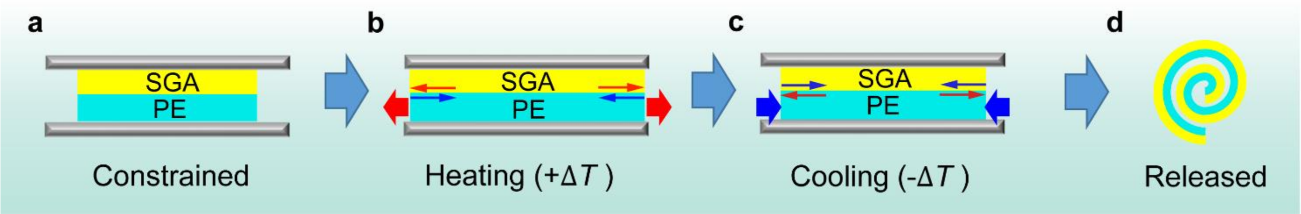

Figure SN1-1. Schematic illustration showing the constrained tempering process of SGA/PE bilayer. (a) An SGA/PE bilayer film sandwiched by two rigid plates. **(b)** Upon heating by ΔT, the PE layer expands laterally, resulting in the tensile force in the SGA layer and compressive force in the PE layer. **(c)** Upon cooling, the PE layer returns to its original configuration while the SGA layer's deformation cannot be fully recovered due to the plastic deformation in step **(b)**, resulting in the residual tensile force in the PE layer and compressive force in the SGA layer. **(d)** After releasing the constraint of the plates, the SGA/PE bilayer film coils due to the internal residual stress.

Theoretical modeling (plane strain assumption) is carried out to predict the curling curvature of the SGA/PE bilayer after constrained tempering, as schematically shown in Fig. SN1-1. In our modeling, the PE layer is assumed as a purely elastic material, while the SGA layer is assumed as a continuum material with asymmetric elastoplastic behavior under tension and compression, namely elastic and ideally plastic under tension and purely elastic under compression as revealed by the MD simulation (see Fig. 4a). Given the negligible thermal expansion of the SGA layer, its deformation in the heating stage (Fig. SN1-1b) mainly results from the stretching by the attached PE layer. For the PE layer, on the other hand, the deformation includes two portions. One is the thermal expansion and the other is the strain caused by the reaction forces (compression) from the SGA layer. Consider a segment of a bilayer strip. The forces experienced are shown in Fig. SN1-2. The perfect bonding condition between the SGA and PE layers implies that

$$\alpha \Delta T\left(1+\nu_{\mathrm{PE}}\right)-\frac{F}{E'_{\mathrm{PE}} b t_{\mathrm{PE}}}=\frac{F}{E'^{\mathrm{t}}_{\mathrm{SGA}} b t_{\mathrm{SGA}}} \qquad \text{(SN1-1)}$$

where ΔT is the temperature increment during heating, F is the lateral interaction force between the SGA and PE layers, $E'_{\mathrm{PE}} = E_{\mathrm{PE}}/(1-\nu_{\mathrm{PE}}^2)$ and $E'^{\mathrm{t}}_{\mathrm{SGA}} = E^{\mathrm{t}}_{\mathrm{SGA}}/(1-\nu_{\mathrm{SGA}}^2)$ with E_{PE}, ν_{PE} being the elastic modulus and Poisson's ratio, α and t_{PE} are the thermal expansion coefficient and thickness of the PE layer respectively, and $E^{\mathrm{t}}_{\mathrm{SGA}}$, ν_{SGA}, t_{SGA} denote the tensile elastic modulus, Poisson's ratio and thickness of the SGA layer respectively, and b is the width of the bilayer strip. The normal stress in the SGA layer along x-direction, if assumed uniform on the cross-section, is given by

$$\sigma_x^{(\mathrm{SGA})} = \frac{F}{bt_{\mathrm{SGA}}} = \frac{\alpha \Delta T \left(1+\nu_{\mathrm{PE}}\right)}{1/E'^{\mathrm{t}}_{\mathrm{SGA}} + t_{\mathrm{SGA}}/t_{\mathrm{PE}}E'_{\mathrm{PE}}} \tag{SN1-2}$$

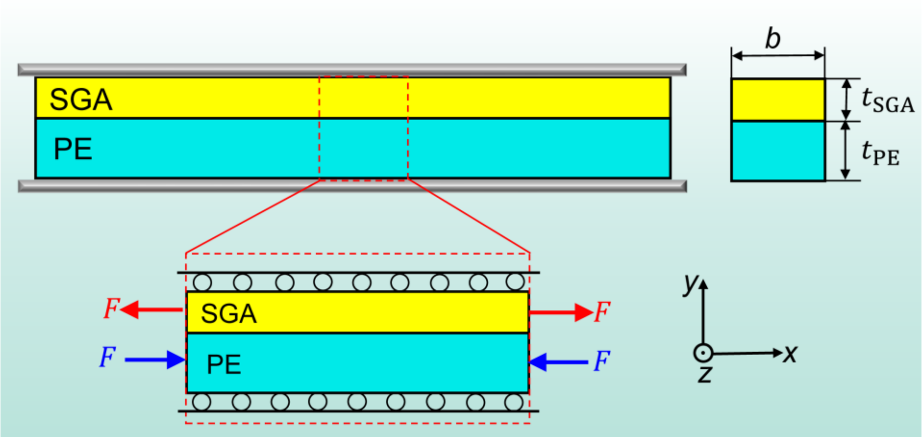

Figure SN1-2. Internal forces experienced by the SGA/PE bilayer under constrained heating.

According to Hooke's law and plane strain condition, it is easy to demonstrate that the stress along z-direction is $\sigma_z^{(\mathrm{SGA})} = \nu_{\mathrm{SGA}}\sigma_x^{(\mathrm{SGA})}$. The constraint applied along y-direction by glass slides mainly functions to restrain the bending deformation of bilayer strip, while the compressive stress exerted is negligible compared with the stresses along x and z directions. In our analysis, therefore, the stress along y-direction is neglected, i.e., $\sigma_y^{(\mathrm{SGA})} = 0$. Based on the von Mises criterion, yielding will not happen until

$$\sigma_{\mathrm{v}} = \sqrt{\frac{1}{2}\left[\left(\sigma_x^{(\mathrm{SGA})} - \sigma_z^{(\mathrm{SGA})}\right)^2 + \left(\sigma_x^{(\mathrm{SGA})} - \sigma_y^{(\mathrm{SGA})}\right)^2 + \left(\sigma_y^{(\mathrm{SGA})} - \sigma_z^{(\mathrm{SGA})}\right)^2\right]}$$
$$= \sigma_x^{(\mathrm{SGA})}\sqrt{1 - \nu_{\mathrm{SGA}} + \nu_{\mathrm{SGA}}^2} = S_{\mathrm{SGA}}^{\mathrm{t}} \quad \text{(SN1-3)}$$

in which $S_{\mathrm{SGA}}^{\mathrm{t}}$ is the yield strength of SGA under tension. Substituting Eq. (SN1-2) into Eq. (SN1-3), the minimum temperature increment causing plastic deformation in the SGA layer is determined as

$$\Delta T^* = \frac{S_{\mathrm{SGA}}^{\mathrm{t}}}{\alpha(1+\nu_{\mathrm{PE}})\sqrt{1-\nu_{\mathrm{SGA}}+\nu_{\mathrm{SGA}}^2}} \cdot \left(\frac{1}{E_{\mathrm{SGA}}'^{\mathrm{t}}} + \frac{t_{\mathrm{SGA}}}{t_{\mathrm{PE}}E_{\mathrm{PE}}'}\right) \quad \text{(SN1-4)}$$

At the critical moment of yielding, *F* saturates at its maximum value, which can be determined by solving

$$\alpha\Delta T^*(1+\nu_{\mathrm{PE}}) - \frac{F_{\max}}{E_{\mathrm{PE}}' b t_{\mathrm{PE}}} = \frac{F_{\max}}{E_{\mathrm{SGA}}'^{\mathrm{t}} b t_{\mathrm{SGA}}} \quad \text{(SN1-5)}$$

When $\Delta T > \Delta T^*$, temperature increment will only cause plastic strain ($\varepsilon_{\mathrm{p}}^{\mathrm{t}}$) in the SGA layer, while *F* remains constant. Therefore, when $\Delta T > \Delta T^*$, the perfect bonding condition implies

$$\alpha\Delta T(1+\nu_{\mathrm{PE}}) - \frac{F_{\max}}{E_{\mathrm{PE}}' b t_{\mathrm{PE}}} = \frac{F_{\max}}{E_{\mathrm{SGA}}'^{\mathrm{t}} b t_{\mathrm{SGA}}} + \varepsilon_{\mathrm{p}}^{\mathrm{t}} \quad (\text{for } \Delta T > \Delta T^*) \quad \text{(SN1-6)}$$

Eqs. (SN1-5) and (SN1-6) imply that the plastic strain $\varepsilon_{\mathrm{p}}^{\mathrm{t}}$ can be written as

$$\varepsilon_{\mathrm{p}}^{\mathrm{t}} = \alpha(1+\nu_{\mathrm{PE}})(\Delta T - \Delta T^*) \quad \text{(SN1-7)}$$

After the heating stage, the temperature then is reduced to the initial value (Fig. SN1-1c). The PE layer contracts and the tensile load applied to the SGA layer gets released. This causes the recovery of the elastic tensile strain in the SGA layer. Since the SGA layer has experienced permanent elongation during the heating stage, contraction of the PE layer would lead to compressive stress in the SGA layer and tensile stress in the PE layer. After removing the external constraint, the strain misfit between the SGA and PE layers, which is equal to the plastic strain ($\varepsilon_{\mathrm{p}}^{\mathrm{t}}$) of the SGA in the heating stage, causes the bilayer to curl with the PE layer being wrapped inside (Fig. SN1-1d). Consider a segment of the bilayer

strip (see Fig. SN1-3). All the forces acting on the cross-section of the bilayer can be equivalently simplified as axial forces F plus a bending moment M (Fig. SN1-3). Perfect bonding along the interface implies that

$$\varepsilon_{\mathrm{p}}^{\mathrm{t}} - \frac{F}{E_{\mathrm{SGA}}^{\prime \mathrm{c}} b t_{\mathrm{SGA}}} = \frac{F}{E_{\mathrm{PE}}^{\prime} b t_{\mathrm{PE}}} \tag{SN1-8}$$

where $E_{\mathrm{SGA}}^{\prime \mathrm{c}} = E_{\mathrm{SGA}}^{\mathrm{c}}/(1-\nu_{\mathrm{SGA}}^2)$ with $E_{\mathrm{SGA}}^{\mathrm{c}}$ being the compressive elastic modulus of the SGA layer. The compressive elastic modulus of the SGA layer is used here since the stress in the SGA layer is compression dominant, which can be verified later. From Eq. (SN1-8), we can determine F as

$$F = \varepsilon_{\mathrm{p}}^{\mathrm{t}} \left(\frac{1}{E_{\mathrm{PE}}^{\prime} b t_{\mathrm{PE}}} + \frac{1}{E_{\mathrm{SGA}}^{\prime \mathrm{c}} b t_{\mathrm{SGA}}} \right)^{-1} \tag{SN1-9}$$

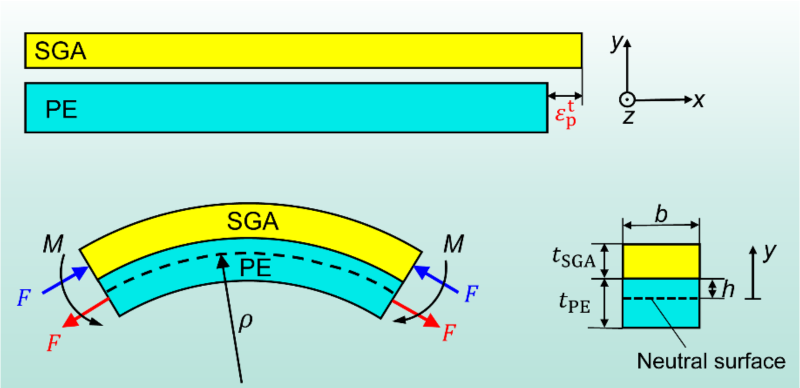

Figure SN1-3. Curling of SGA/PE bilayer after constrained tempering and releasing.

Denote the radius of the curvature of the neutral surface as ρ, as shown in Fig. SN1-3. The bending-induced strain is given by

$$\varepsilon_{x\text{-bend}} = \frac{y}{\rho} \quad (h - t_{\mathrm{PE}} \le y \le h + t_{\mathrm{SGA}}) \tag{SN1-10}$$

where h is the distance from the SGA/PE interface to the neutral surface. The stresses along lateral direction caused by axial force and bending moment in both SGA and PE layers are given by

$$\sigma_x^{(\mathrm{SGA})} = \sigma_{x\text{-bend}}^{(\mathrm{SGA})} + \sigma_{x\text{-axial}}^{(\mathrm{SGA})} = E_{\mathrm{SGA}}^{\prime \mathrm{c}} \frac{y}{\rho} - \frac{F}{b t_{\mathrm{SGA}}} \quad (h \le y \le h + t_{\mathrm{SGA}}) \tag{SN1-11a}$$

$$\sigma_x^{(\mathrm{PE})} = \sigma_{x-\mathrm{bend}}^{(\mathrm{PE})} + \sigma_{x-\mathrm{axial}}^{(\mathrm{PE})} = E'_{\mathrm{PE}}\frac{y}{\rho} + \frac{F}{bt_{\mathrm{PE}}} \qquad (h - t_{\mathrm{PE}} \le y \le h) \tag{SN1-11b}$$

In Eq. (SN1-11a), the whole SGA layer is assumed under compression along the lateral direction. This point will be verified later.

The resultant force on the entire cross-section of the bilayer is zero, which implies

$$\int_h^{h+t_{\mathrm{SGA}}} \sigma_x^{(\mathrm{SGA})} b dy + \int_{h-t_{\mathrm{PE}}}^{h} \sigma_x^{(\mathrm{PE})} b dy = 0 \tag{SN1-12}$$

Substituting Eq. (SN1-11) into Eq. (SN1-12) determines h as

$$h = \frac{1}{2} \cdot \frac{E'_{\mathrm{PE}} t_{\mathrm{PE}}^2 - E'^{\mathrm{c}}_{\mathrm{SGA}} t_{\mathrm{SGA}}^2}{E'_{\mathrm{PE}} t_{\mathrm{PE}} + E'^{\mathrm{c}}_{\mathrm{SGA}} t_{\mathrm{SGA}}} \tag{SN1-13}$$

The resultant moment on the entire cross-section is also zero, giving rise to

$$\int_h^{h+t_{\mathrm{SGA}}} \sigma_x^{(\mathrm{SGA})} y dy + \int_{h-t_{\mathrm{PE}}}^{h} \sigma_x^{(\mathrm{PE})} y dy = 0 \tag{SN1-14}$$

Substituting Eq. (SN1-11) into Eq. (SN1-14) yields

$$\frac{E'^{\mathrm{c}}_{\mathrm{SGA}}}{3\rho}\left[(h + t_{\mathrm{SGA}})^3 - h^3\right] + \frac{E'_{\mathrm{PE}}}{3\rho}\left[h^3 - (h - t_{\mathrm{PE}})^3\right] = \frac{F}{2b}(t_{\mathrm{PE}} + t_{\mathrm{SGA}}) \tag{SN1-15}$$

Combining Eqs. (SN1-7) (SN1-9) (SN1-13) and (SN1-15) gives the curvature of the bilayer strip, κ, as

$$\kappa = \frac{1}{\rho} = \frac{6\alpha(1 + \nu_{\mathrm{PE}})(\Delta T - \Delta T^*)(t_{\mathrm{PE}} + t_{\mathrm{SGA}})}{4(t_{\mathrm{PE}}^2 + t_{\mathrm{SGA}}^2) + 6 t_{\mathrm{SGA}} t_{\mathrm{PE}} + \frac{E'_{\mathrm{PE}} t_{\mathrm{PE}}^3}{E'^{\mathrm{c}}_{\mathrm{SGA}} t_{\mathrm{SGA}}} + \frac{E'^{\mathrm{c}}_{\mathrm{SGA}} t_{\mathrm{SGA}}^3}{E'_{\mathrm{PE}} t_{\mathrm{PE}}}} \tag{SN1-16}$$

Considering $t_{\mathrm{SGA}} \ll t_{\mathrm{PE}}$, an approximation of Eq. (SN1-16) is given by

$$\kappa \approx \frac{6\alpha(1 + \nu_{\mathrm{PE}})(\Delta T - \Delta T^*)}{4 t_{\mathrm{PE}} + E'_{\mathrm{PE}} t_{\mathrm{PE}}^2 / E'^{\mathrm{c}}_{\mathrm{SGA}} t_{\mathrm{SGA}}}. \tag{SN1-17}$$

The prediction of the curvature of the SGA/PE bilayer above is based on the assumption that the whole SGA layer is under compression along the lateral direction. This, according to Eq. (SN1-11a), requires

that

$$\sigma_x^{(\mathrm{SGA})}\Big|_{y=h+t_{\mathrm{SGA}}} = E_{\mathrm{SGA}}^{\prime\mathrm{c}} \frac{h+t_{\mathrm{SGA}}}{\rho} - \frac{F}{bt_{\mathrm{SGA}}} < 0 \tag{SN1-18}$$

Recalling Eqs. (SN1-9) and (SN1-16), Eq. (SN1-18) can be rewritten as

$$4\left(t_{\mathrm{PE}}^2 + t_{\mathrm{SGA}}^2\right) + 6t_{\mathrm{SGA}}t_{\mathrm{PE}} + \frac{E'_{\mathrm{PE}}t_{\mathrm{PE}}^3}{E_{\mathrm{SGA}}^{\prime\mathrm{c}}t_{\mathrm{SGA}}} + \frac{E_{\mathrm{SGA}}^{\prime\mathrm{c}}t_{\mathrm{SGA}}^3}{E'_{\mathrm{PE}}t_{\mathrm{PE}}} - 6\left(t_{\mathrm{PE}} + t_{\mathrm{SGA}}\right)\left(h + t_{\mathrm{SGA}}\right)\left(1 + \frac{E_{\mathrm{SGA}}^{\prime\mathrm{c}}t_{\mathrm{SGA}}}{E'_{\mathrm{PE}}t_{\mathrm{PE}}}\right) > 0 \tag{SN1-19}$$

Taking $E_{\mathrm{SGA}}^{\mathrm{c}} = 2.2$ GPa, $\nu_{\mathrm{SGA}} = 0.19$, $E_{\mathrm{PE}} = 300$ MPa, $\nu_{\mathrm{PE}} = 0.46$ and $t_{\mathrm{PE}} = 10$ μm, the satisfaction of the above inequality requires $t_{\mathrm{SGA}} \le 2.2$ μm. In the present work, this condition is well satisfied as the thickness of our SGA ranges from 0.1 to 0.6 μm

Supplementary Note 2. Kinematic analysis of the light-driven rolling motor (SGA/PE roll)

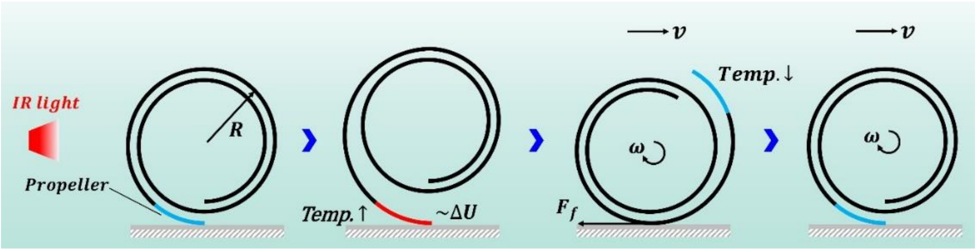

Figure SN2-1. Snapshots of the rolling process of an SGA/PE bilayer motor.

The rolling process of an SGA/PE roll-based motor under IR light heating is schematically depicted in Fig. SN2-1. Set the initial point of time as the onset moment of rolling. Consider a time period from 0 to *t*. Energy conservation implies that

$$U_{\mathrm{total}} + W_{\mathrm{f}} = E_{\mathrm{k}} \quad \text{(SN2-1)}$$

where U_{total} refers to the total strain energy released by the SGA/PE bilayer due to unfolding, W_{f} is the work done by the friction force and E_{k} stands for the increment of the kinetic energy. The total released strain energy can be expressed as $U_{\mathrm{total}} = n\Delta U$, where n is the number of times that the propeller pushes the ground; ΔU is the average strain energy released from each touch; W_{f} can be described by $W_{\mathrm{f}} = -F_{\mathrm{f}} \int_0^t v dt$, where F_{f} is the rolling friction force between the roller and the ground. Assume that the roller is a thin-walled cylinder. The kinetic energy can be expressed as $E_{\mathrm{k}} = mv^2$, where *m* stands for the mass of the roller.

For a curled bilayer film, the stored strain energy is proportional to κ^2 as the previous theory indicates[63], where κ is the curvature. First-order approximation indicates that

$$\frac{\Delta U}{U_0 - U_{\mathrm{S}}} \propto \Delta\kappa$$

where U_0 is the initial strain energy stored, U_S is the remaining strain energy after a sufficiently long time of heating by the IR light, ΔU is the change of strain energy near the initial state, and $\Delta\kappa$ is the change of curvature near the initial curled configuration.

The above discussion on the prediction of curvature indicates that $\Delta\kappa \propto \Delta T$, where ΔT refers to the temperature increase. To predict the temperature change in the SGA/PE bilayer due to IR light illustration, a thermal equilibrium equation is established as follows

$$QA + hA(T_e - T) = cm\frac{dT}{dt}$$

where Q is a constant representing the energy reception rate from the IR light on a unit area, A is the effective area that is exposed upon the IR light, T_e is the environmental temperature, h and c represent the surface heat transfer coefficient and heat capacity of the material, respectively. The solution of the above equation indicates that

$$\Delta T \propto 1 - e^{-k\tau_c}$$

where constant $k = \frac{hA}{cm}$ and τ_c represents the exposure time in the IR light which is inversely proportional to the speed of the roller, namely $\tau_c \propto \frac{1}{v}$. We therefore have

$$\frac{\Delta U}{U_0 - U_S} \propto 1 - e^{-\frac{k'}{v}}$$

Considering $n = \frac{\int_0^t v dt}{2\pi R}$ and $F_f = \mu mg$ (μ is the rolling friction coefficient, m and R are the total mass and radius of the roller, respectively), the substitution of the above scaling law into Eq. (SN2-1) gives the governing equation about velocity v as

$$\left(1 - e^{-\frac{k'}{v}}\right)(U_0 - U_S)\frac{\int_0^t v dt}{2\pi R} - \mu mg \int_0^t v dt = mv^2 \quad \text{(SN2-2)}$$

For the SGA/PE bilayer, we take $m = 2.1 \times 10^{-4}$ kg, $R = 1 \times 10^{-3}$ m, $U_0 = 2.38 \times 10^{-6}$ J, $U_S = 3.96 \times 10^{-7}$ J. The values of U_0 and U_S are calculated by an established theory[63] with the

corresponding curvatures $\kappa_0 = 2.2 \times 10^{-3}\ \mathrm{m}^{-1}$ and $\kappa_\mathrm{S} = 0.9 \times 10^{-3}\ \mathrm{m}^{-1}$, respectively. Runge-Kutta method is applied to solve Eq. (SN2-2) numerically for velocity v. By best fitting ($R^2 = 0.971$) the numerical results with the experimental data points, parameters μ and k' can be determined as μ =0.148, $k' = 0.24$ respectively, as shown in Fig. 7c.

It is found that the velocity of the roller increases monotonically with the time and then asymptotically approaches to a limited value denoted by v_f as $t \to \infty$. By taking the derivative on both sides of Eq. (SN2-2) with respect to t and assuming v as a constant, an equation for determining v_f can be obtained as follows

$$\left(1 - \mathrm{e}^{-\frac{k'}{v_\mathrm{f}}}\right)(U_0 - U_\mathrm{S}) - 2\pi R \cdot \mu m g = 0 \tag{SN2-3}$$

Solving Eq. (SN2-3) for v_f gives rise to

$$v_\mathrm{f} = \frac{-k'}{\ln\left(1 - \frac{2\pi R \cdot \mu m g}{U_0 - U_\mathrm{S}}\right)} \tag{SN2-4}$$

Taking $m = 2.1 \times 10^{-4}$ kg, $R = 1 \times 10^{-3}$ m, $U_0 = 2.38 \times 10^{-6}$ J, $U_\mathrm{S} = 3.96 \times 10^{-7}$ J, μ =0.148, $k' = 0.24$, $g = 9.8$ N kg^{-1} in Eq. (SN2-4), the upper limit of velocity of such motor is estimated to be 7.19 cm s^{-1}, as displayed by the black dash line in Fig. 7c.

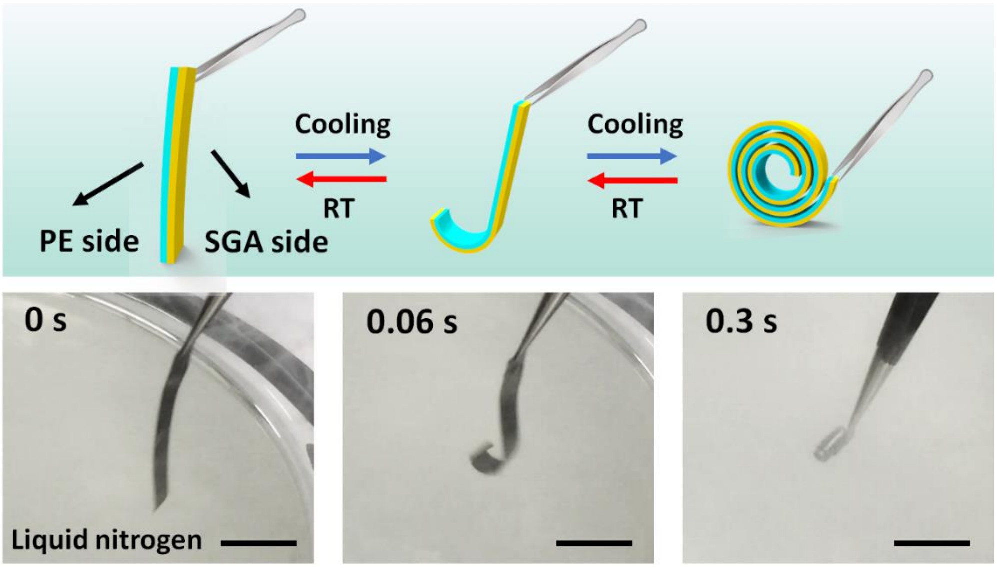

Supplementary Figure 1. Actuation behavior of SGA/PE bilayer upon cooling by liquid nitrogen. Scale bars, 1 cm.

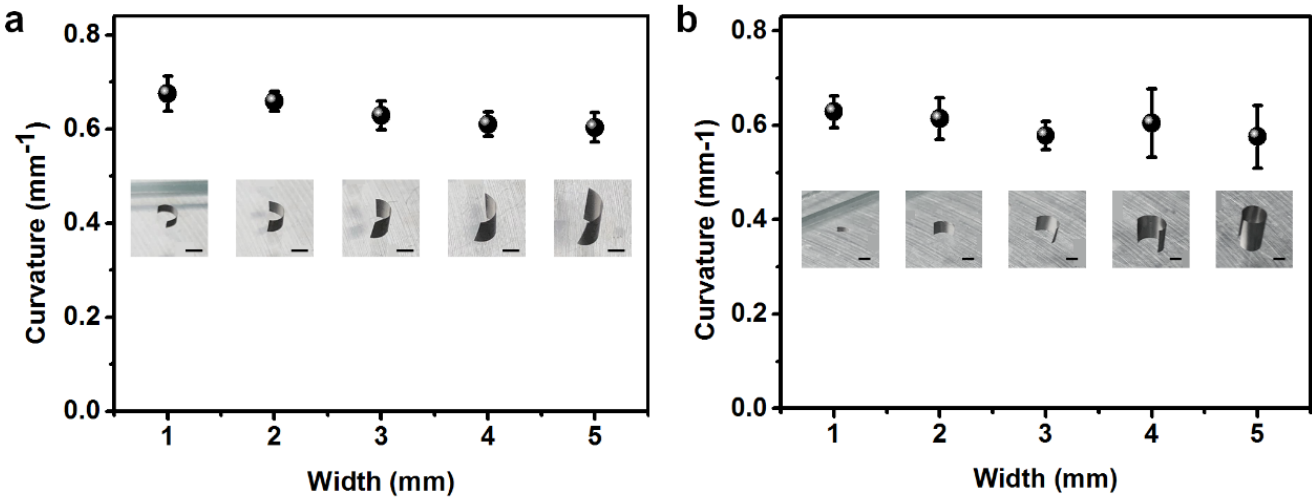

Supplementary Figure 2. Actuation performance of the as-prepared SGA/PE bilayers with different sizes. (**a**) The coiling curvature of the samples with different widths and the same length of 5 mm. (**b**) The coiling curvature of the samples with different widths and the same aspect ratio of 2. The SGA ply number in all samples is 3 and the actuating temperature is ΔT=15 °C. Error bars represent standard deviations. Scale bar: 2 mm.

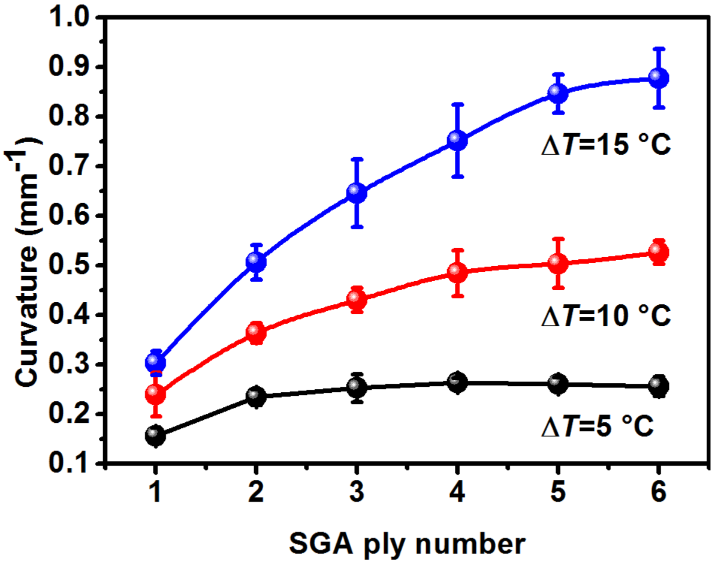

Supplementary Figure 3. Actuation performance of as-prepared SGA/PE bilayers with varied SGA ply number upon different actuation temperatures. Error bars represent standard deviations.

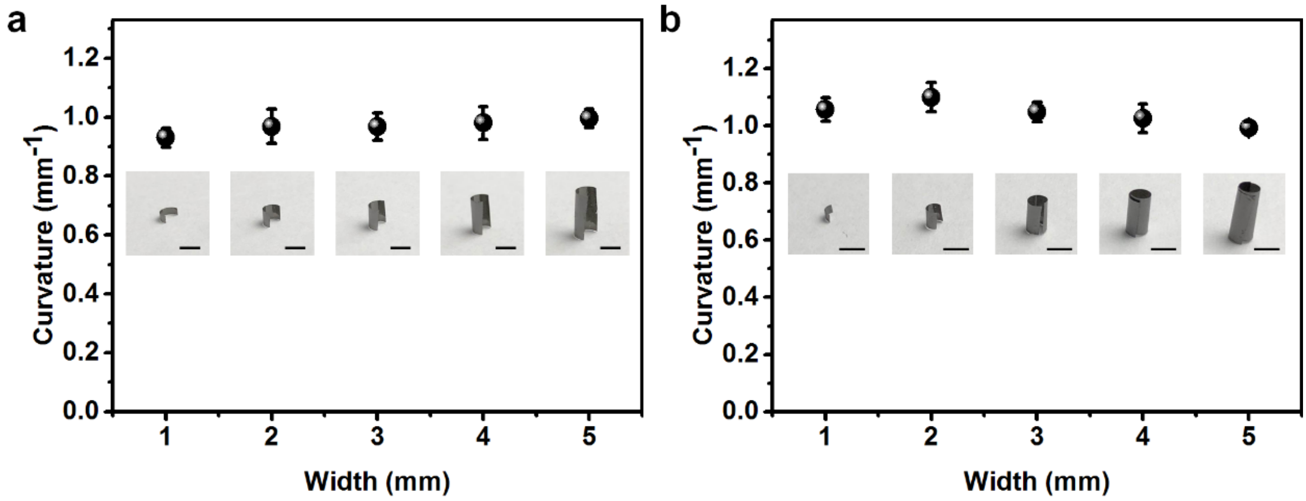

Supplementary Figure 4. The morphing behavior of tempered SGA/PE films with different sizes. (**a**) The curvature of tempered samples with different widths while the same length of 5 mm. (**b**) The curvature of tempered samples with different widths while the same aspect ratio of 2. The SGA ply number in all samples is 3 and the tempering temperature is ΔT=30 °C. Error bars represent standard deviations. Scale bar: 2 mm.

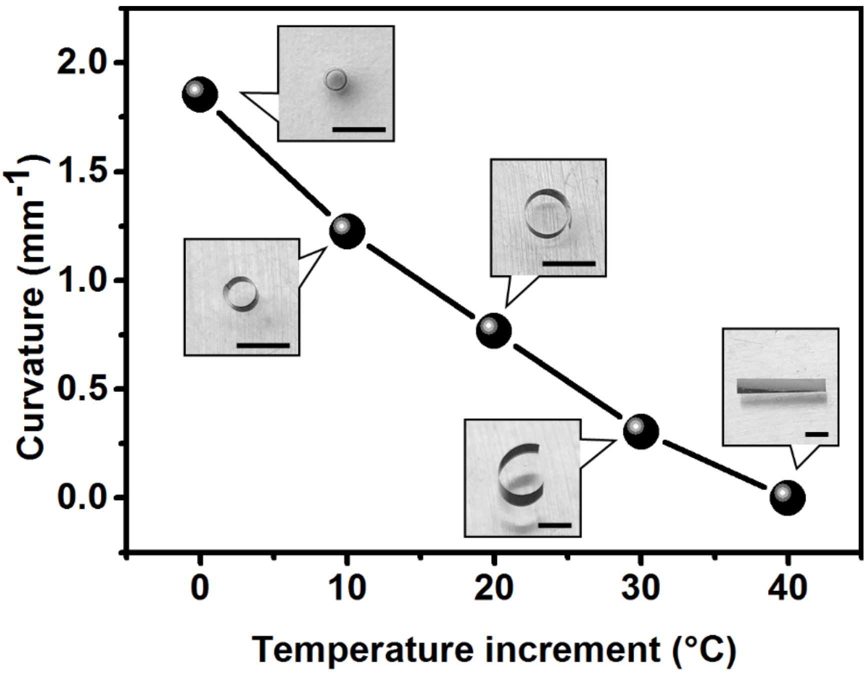

Supplementary Figure 5. Variation of the curvature of a tempered SGA/PE film with actuating temperature. Scale bar: 3 mm.

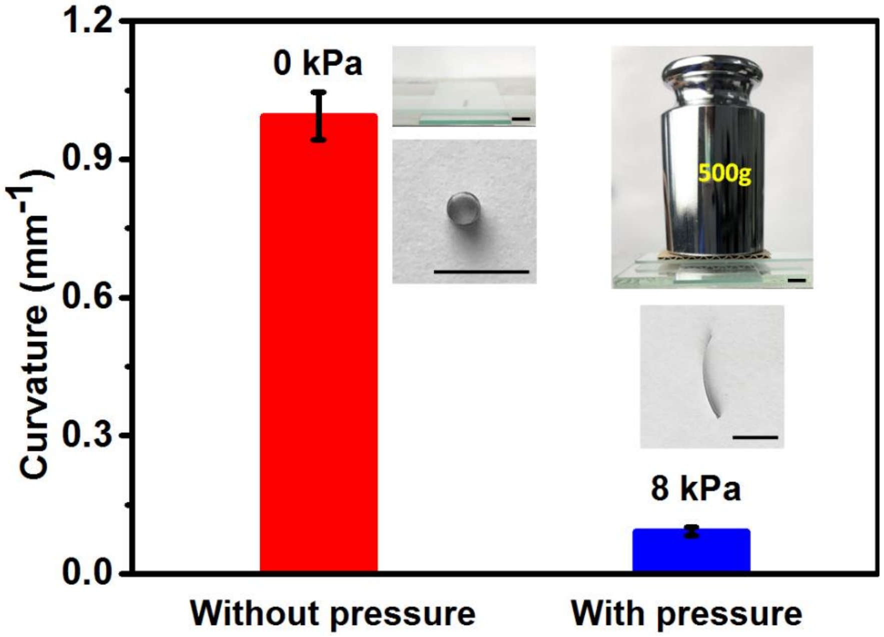

Supplementary Figure 6. Comparison of the configurations of SGA/PE films after tempering with and without extra vertical pressure. The SGA ply number is 3 and the tempering temperature is ΔT=30 °C. Error bars represent standard deviations. Scale bar: 5 mm.

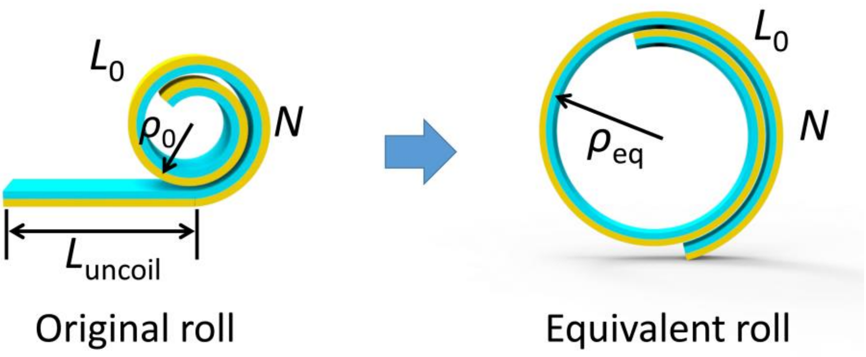

Supplementary Figure 7. Schematic illustration showing the definition of equivalent curvature of a roll in the uncoiling process. To quantify the coiling extend of a partially coiled roll in the uncoiling process, an equivalent fully coiled roll is introduced. The equivalent roll is assumed to have the same total length (L_0) and number of turns (N) as the original roll. The curvature of the equivalent roll is $\kappa_{eq} = (\rho_{eq})^{-1} = \left(\frac{L_0}{2\pi N}\right)^{-1} = \left(\frac{\rho_0 L_0}{L_0 - L_{uncoil}}\right)^{-1} = (1 - L_{uncoil}/L_0)\kappa_0$, where L_{uncoil} is the length of the uncoiled portion and $\kappa_0 = 1/\rho_0$ is the curvature of the coiled portion.

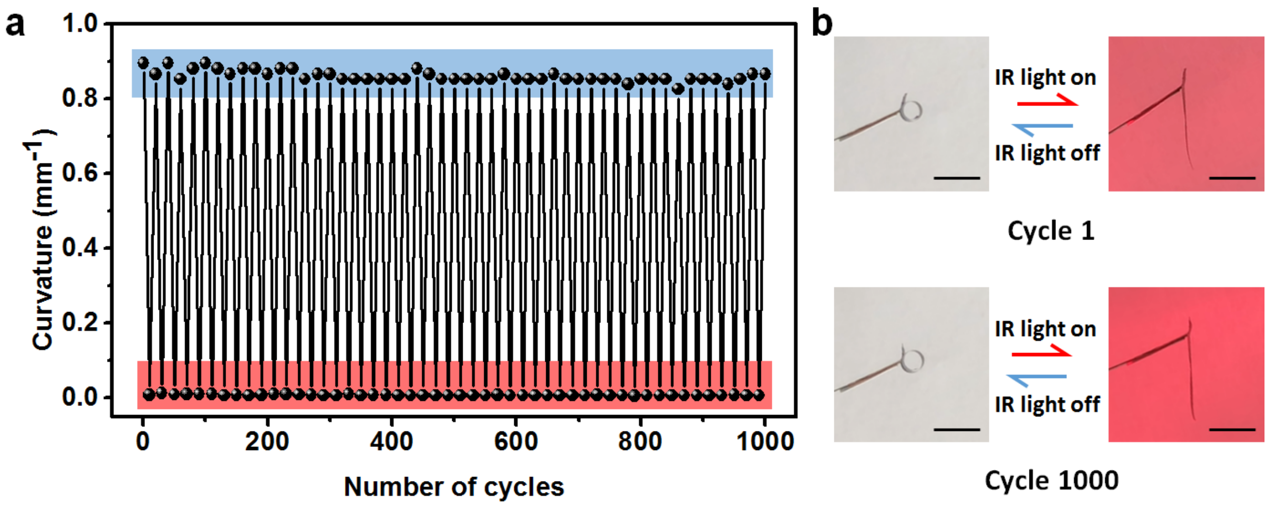

Supplementary Figure 8. Stability test of the SGA/PE film upon cyclic IR light illumination. (**a**) The curvatures of the film before and after 20 cycles of unrolling-rolling actuation. (**b**) Optical images showing the morphologies of the film after cycle 1 and cycle 1000, respectively. The irradiation intensity of the IR light is 120 mW cm^{-2}. Scale bars: 5 mm.

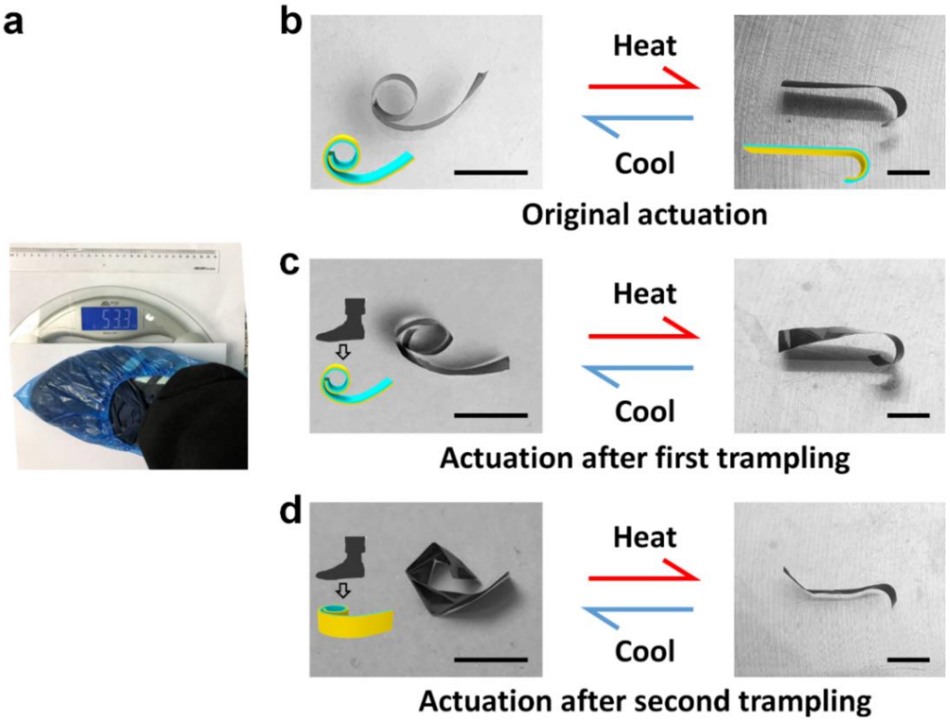

Supplementary Figure 9. Robustness of the SGA/PE film actuators. (**a**) Optical images showing the actuator being trampled by an adult (~53 kg). (**b**) Actuation performance of the intact SGA/PE film, (**c**) actuation performance after the first trampling along the film thickness direction, and (**d**) actuation performance after the second trampling along the side direction of a tempered SGA/PE actuator. Scale bar: 5 mm.

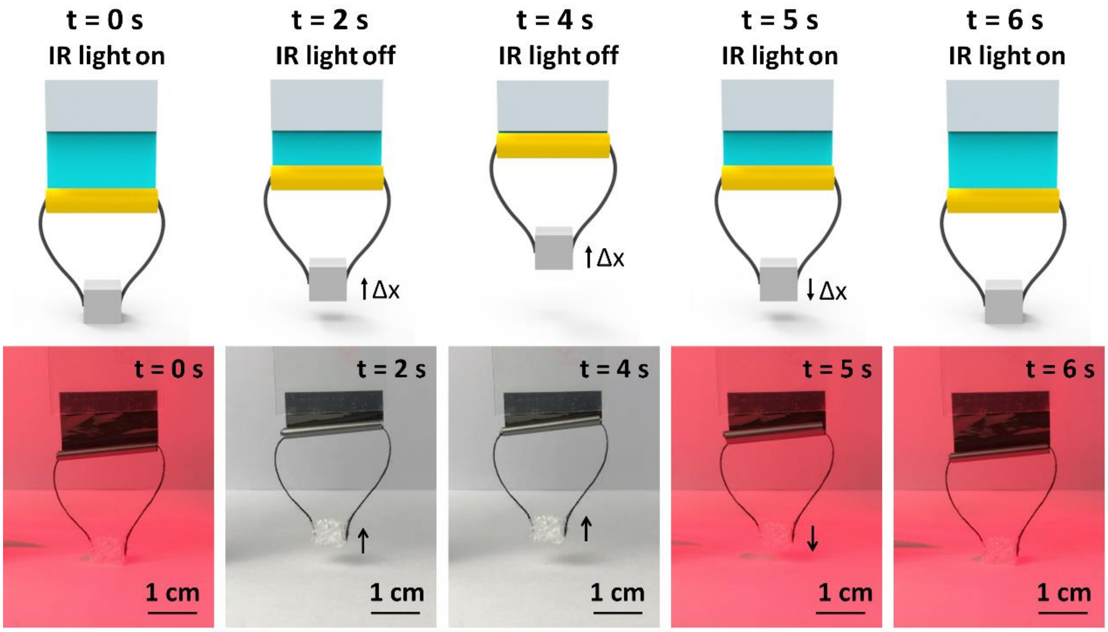

Supplementary Figure 10. Schematic illustration and snapshots of the weightlifting experiment under controlled IR light illumination. The load is ten times of the actuator weight. Vertical displacement is 8 mm. The actuator here is SGA (6 plies)/PE tempered by ΔT = 50 °C. The intensity of IR light illumination 120 mW cm^{-2}.

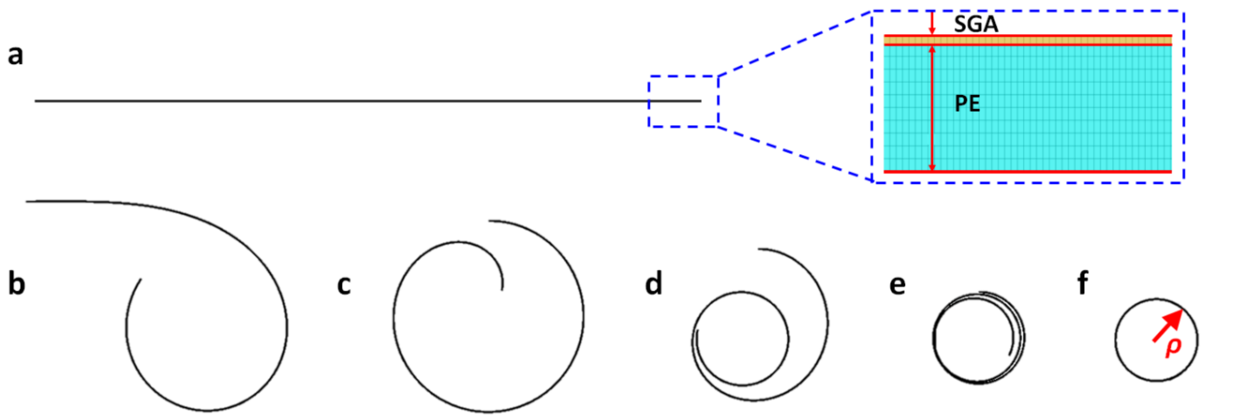

Supplementary Figure 11. Simulated deforming process of a tempered SGA/PE bilayer film after being released from external constraint. (**a**) Configuration of an SGA/PE bilayer film after constrained tempering. (**b-f**) Snapshots of the deforming process of the tempered SGA/PE bilayer after removing the constraint. Parameters adopted in the simulation: t_{SGA}=0.6 μm, ΔT = 40 °C.

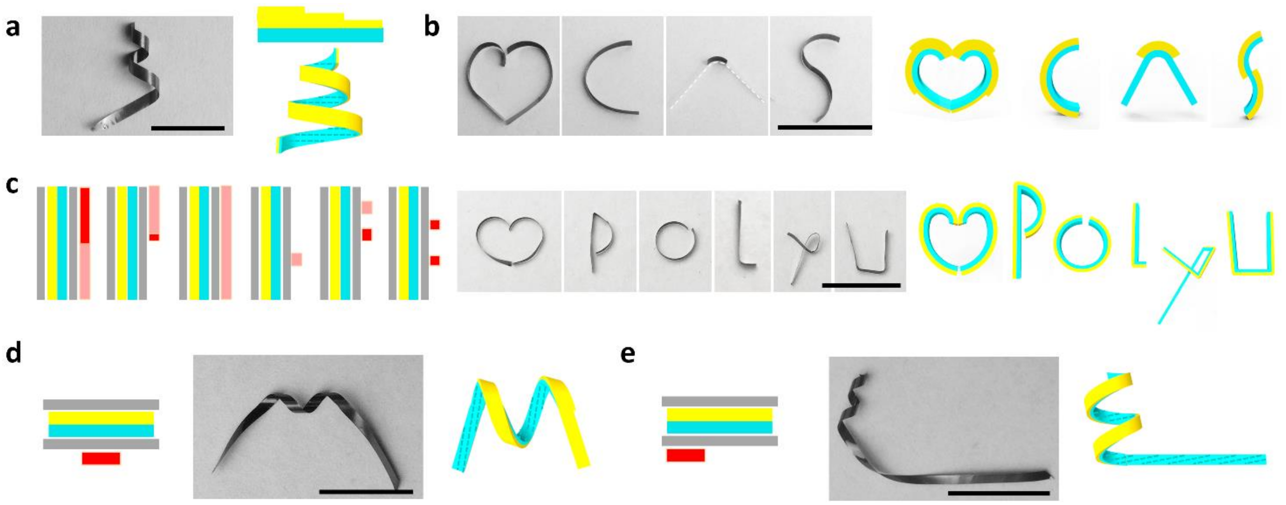

Supplementary Figure 12. Complex configurations of SGA/PE films programmed by mixed strategies. (**a**) 3D conical helical structure achieved by non-uniform SGA plus bias-cut PE in which the long edge is oblique to its alignment direction. (**b-c**) Slogans made of SGA/PE films with configurations programmed by multiple strategies including non-uniform SGA, localized/alternate SGA patches, localized/non-uniform tempering and so on. (**d,e**) Centralized and biased helical structures achieved by localized tempering and bias-cut PE. The dash lines in the schematics of A, D, and E represent the alignment direction of the PE layer. Scale bars: 1 cm.

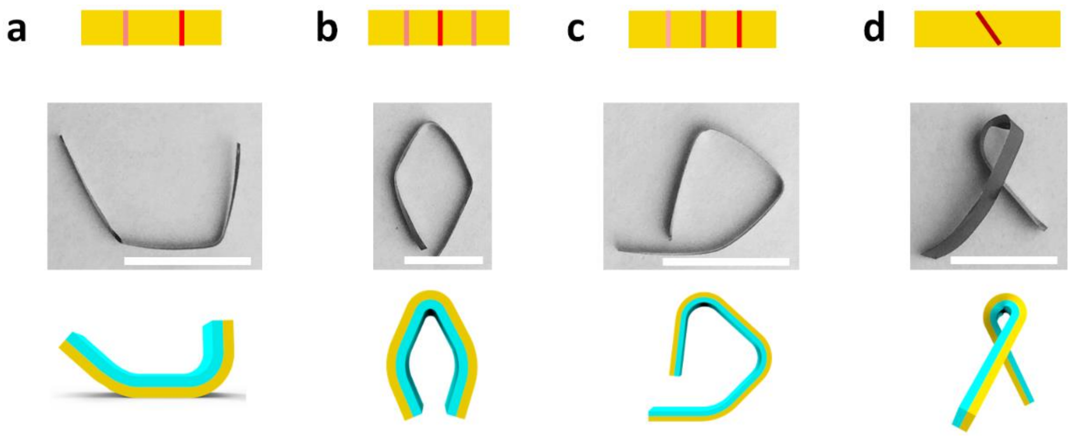

Supplementary Figure 13. Programmed configurations of SGA/PE bilayer films through constrained tempering with Direct Laser Writing technique. Laser writing patterns are schematically illustrated by red lines with shade representing the irradiation time. (**a**) U shape, (**b**) Diamond shape, (**c**) Cook shape, and (**d**) Red Ribbon shape. Scale bars, 1 cm.

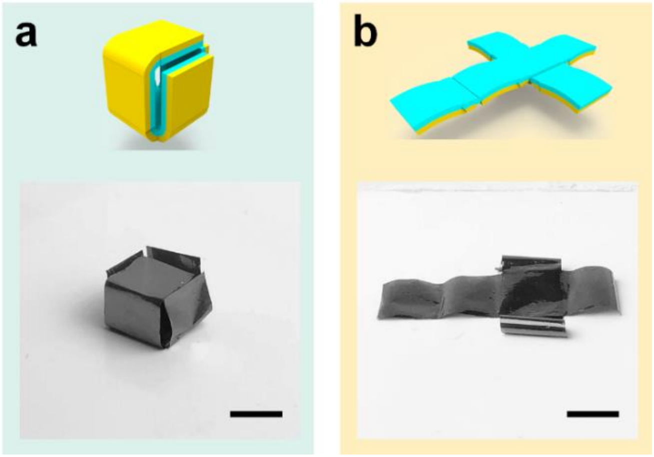

Supplementary Figure 14. The tempered SGA/PE film exhibits programmed cubic configuration at ambient temperature (a) and unfolds upon heating (ΔT = 10 °C) (b). Scale bar: 10 mm.

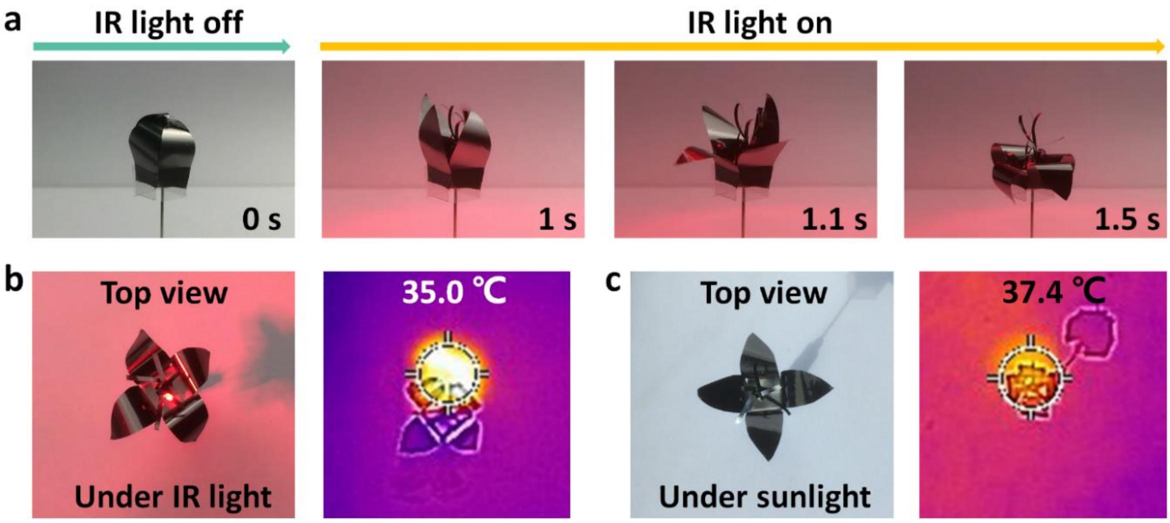

Supplementary Figure 15. An artificial water lily assembled from curved units of SGA/PE bilayer. (**a**) The blooming process of water lily triggered by IR light illumination. (**b, c**) Optical and infrared images of the bloomy water lily under IR light illumination and sunlight illumination.

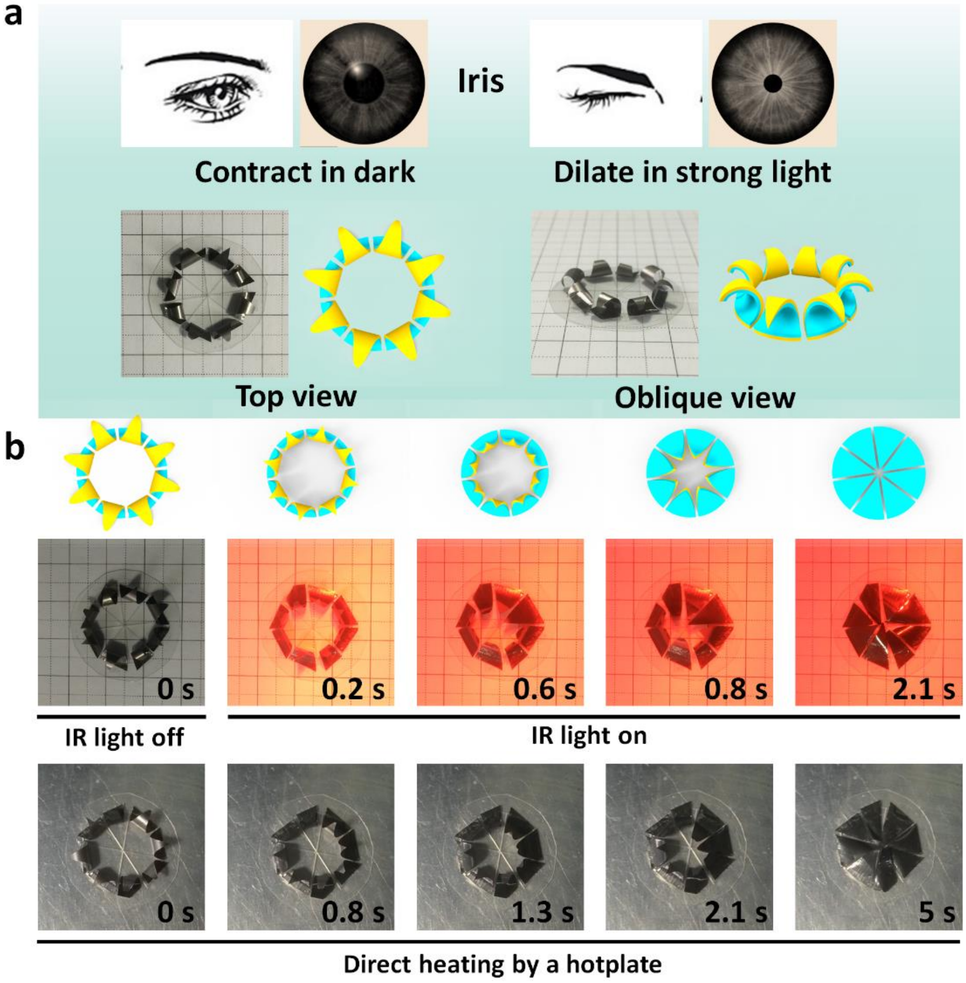

Supplementary Figure 16. An artificial iris assembled from curved units of SGA/PE bilayer. (**a**) An artificial iris to mimic the natural iris which contracts in dark and dilates in strong light. (**b**) Dilatation of the artificial iris upon IR light illumination and direct heating by hotplate.

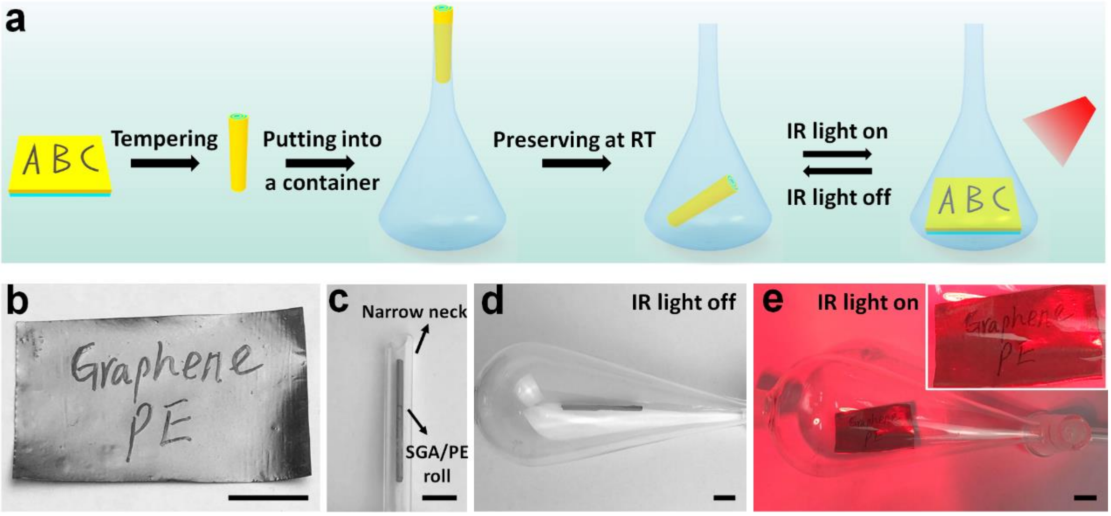

Supplementary Figure 17. Tempered SGA/PE bilayers for information storage, encryption, and decryption. (**a**) Schematic illustration showing a message concealed in a coiled SGA/PE bilayer roll. The thin roll can then be put into a container with a narrow neck for storage. The message can be reversibly displayed and hidden upon the application of external IR light illumination. (**b**) Optical image of an as-prepared flat SGA/PE film with a message written on the SGA side. (**c**) The coiled SGA/PE roll after constrained tempering can pass through a narrow neck of a container. Reversible encryption (**d**) and decryption (**e**) of the message under controlled IR light illumination. Scale bars, 1 cm.

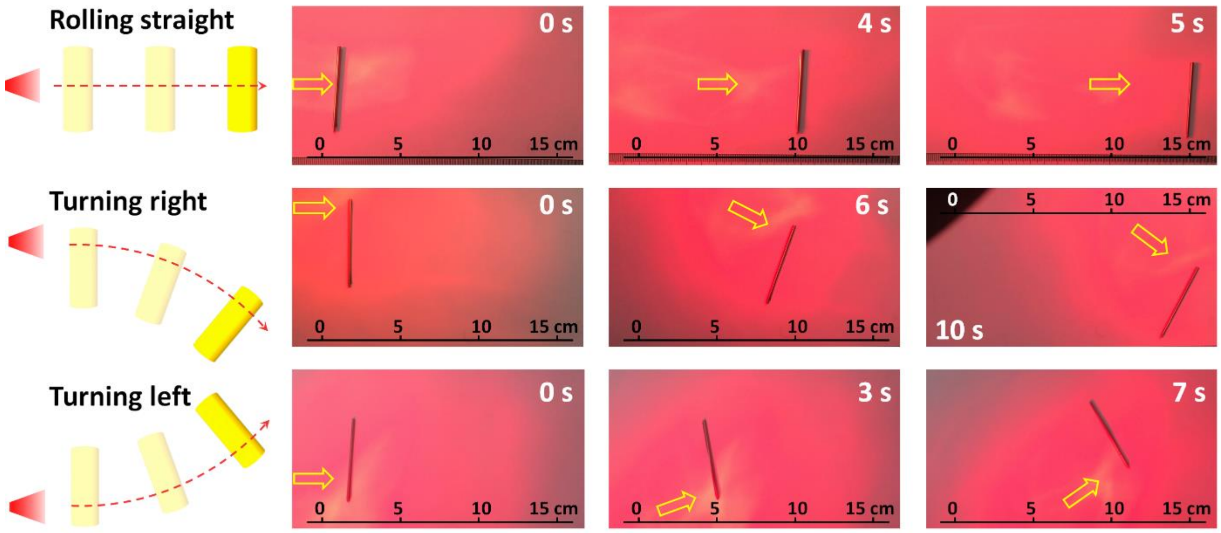

Supplementary Figure 18. Three basic locomotion modes of the light-driven motor (SGA/PE roll).

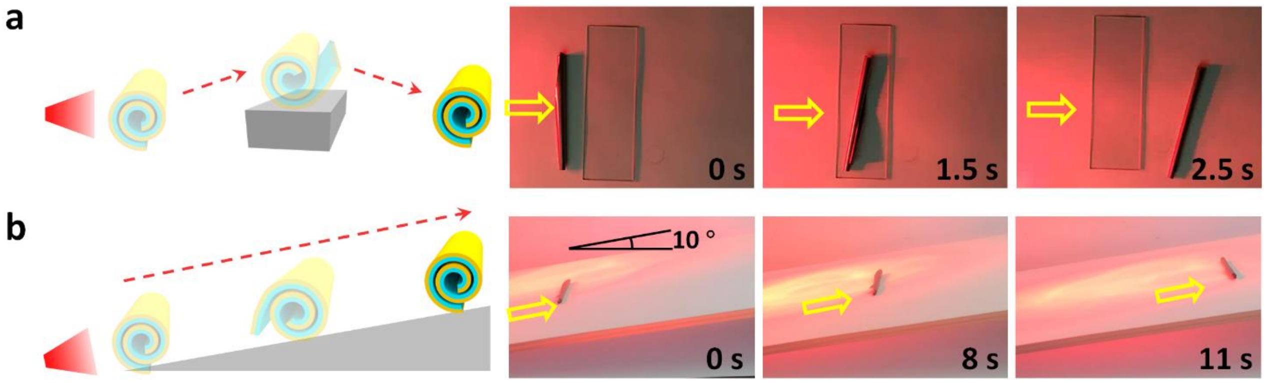

Supplementary Figure 19. Snapshots of a rolling motor crossing obstacles (glass slide) (a) and climbing ramp (10°) (b).

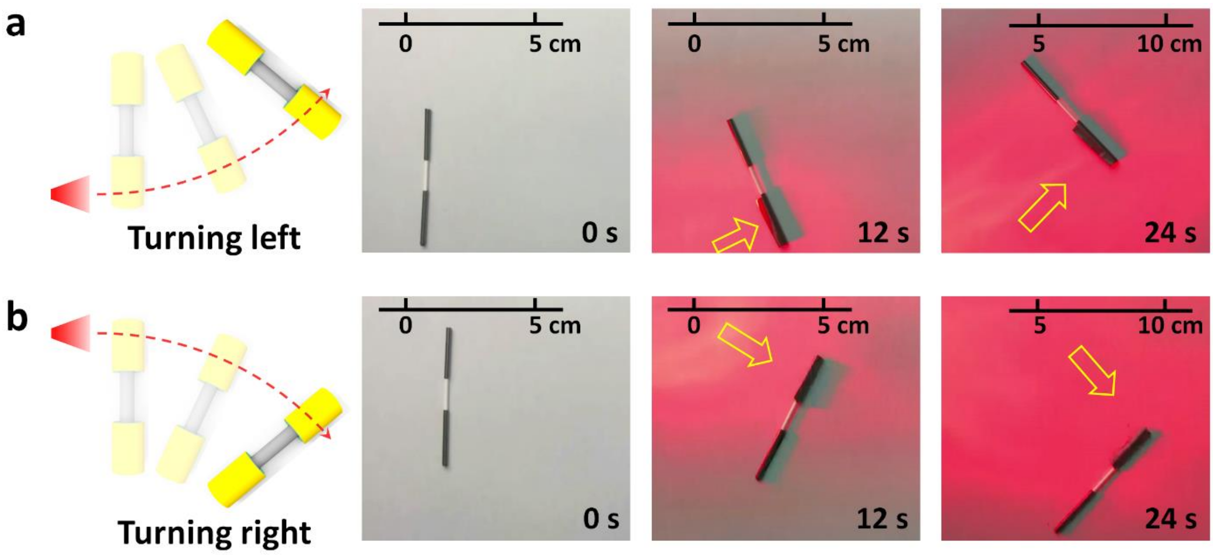

Supplementary Figure 20. Turning motions of a bi-wheel motor achieved by controlling the position of illumination of lateral IR light. (**a**) Turning left motion upon IR light illumination on the right side of the bi-wheel motor. (**b**) Turning right motion upon IR light illumination on the left side of the bi-wheel motor.

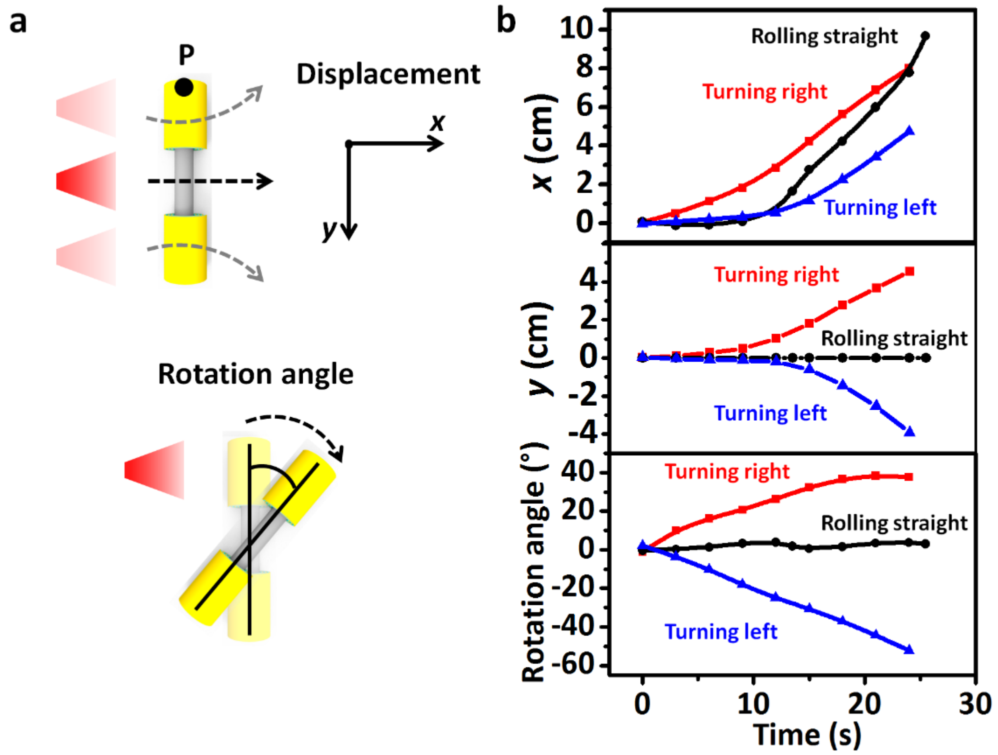

Supplementary Figure 21. Characterization of the controlled locomotion directions of a bi-wheel motor. (**a**) Schematic illustration of the direction of displacement and rotation angel. (**b**) Displacement in *x*-direction, displacement in *y*-direction (lateral direction) and rotation angle versus time curves. Here, the displacement is for the point *P* as indicated in (a).

Supplementary Table 1. Comparison of actuation performance between our SGA/PE roll and other similar actuators reported in the literatures

Materials	Thickness /mm	Curvature /mm^{-1}	Thickness ×Curvature	Time/s	Reference
SGA/PE	0.013	2.53	0.0329	0.37	This work
PFSA	0.075	0.31	0.0233	0.25	(16)
LCP	0.014	0.23	0.0032	0.38	(55)
PVDF	0.003	3.2	0.0096	0.4	(17)
Porous polymer	0.03	1.33	0.0399	0.4	(19)
SGO/PVDF	0.005	2.2	0.011	1	(6)
RGO-CNT/PDMS	0.122	0.7	0.0854	3.6	(58)
CNT/PDMS	0.105	0.24	0.0252	4.86	(56)
PEO/PI	0.09	0.11	0.0099	8	(44)
SACNT/BOPP	0.047	0.103	0.0048	10	(59)
CNT/BOPP	0.075	0.16	0.012	10	(14)
PEDOT:PSS/PDMS	0.121	0.3	0.0362	10	(57)
LDPE/PVC	0.04	0.25	0.01	40	(54)
