# Supplementary material for: Asymmetric elastoplasticity of stacked graphene assembly actualizes programmable untethered soft robotics
Source: Nat Commun. 2020 Aug 31;11:4359. doi: 10.1038/s41467-020-18214-0 (PMC7459344; doi:10.1038/s41467-020-18214-0)
Supplement: Supplementary file 3 — Description of Additional Supplementary Files [file 41467_2020_18214_MOESM3_ESM.docx]

Description of Additional Supplementary Files

Title: Supplementary Movie 1.

Description: Cyclic actuation test

Title: Supplementary Movie 2.

Description: Deformation process of an SGA film under tension and compression as calculated by molecular dynamics simulation

Title: Supplementary Movie 3.

Description: Shape evolution of a tempered SGA/PE bilayer after being released from external constraint as calculated by finite element analysis

Title: Supplementary Movie 4.

Description: An artificial water lily

Title: Supplementary Movie 5.

Description: An artificial iris

Title: Supplementary Movie 6.

Description: A sit-up robot

Title: Supplementary Movie 7.

Description: Close-up shot of the rolling process of a light-driven motor (SGA/PE roll)

Title: Supplementary Movie 8.

Description: Locomotion of a light-driven motor (SGA/PE roll) under different grounds

Title: Supplementary Movie 9.

Description: Controlled locomotion of a light-driven motor (SGA/PE roll) to push a toy football into a goal

Title: Supplementary Movie 10.

Description: Loading, transporting, unloading of cargo by an SGA/PE roll under controlled IR light illumination

Title: Supplementary Movie 11.

Description: Three locomotion modes of a bi-wheel motor assembled from SGA/PE rolls

Title: Supplementary Movie 12.

Description: A four-wheel chassis system by parallel assembly of two bi-wheel motors

Title: Supplementary Movie 13.

Description: A four-wheel truck produced by parallel assembly of SGA/PE rolls
